# Custom Bone Regeneration (CBR): An Alternative Method of Bone Augmentation—A Case Series Study

**DOI:** 10.3390/jcm11164739

**Published:** 2022-08-13

**Authors:** Daniele De Santis, Luciano Umberto, Donadello Dario, Faccioni Paolo, Morris Zarantonello, Cristian Alberti, Giuseppe Verlato, Federico Gelpi

**Affiliations:** 1 Head and Neck Department, Department of Surgery, Dentistry, Pediatrics and Gynecology, University of Verona, 37124 Verona, Italy; 2 Free Practitioner, 36100 Vicenza, Italy; 3 Department of Diagnostics, Public Health University of Verona, 36100 Verona, Italy

**Keywords:** custom bone regeneration, bone defect, digital flow, superimposition, digital planning in surgery

## Abstract

We performed this clinical study in order to evaluate the reliability of the Guided Bone Regeneration (GBR) surgical technique through the use of customized CAD CAM titanium meshes (Yxoss CBR^®^ Reoss) in order to show an alternative method of bone augmentation. Materials and methods: Nine patients presenting 10 bone defects were referred to solve oral dysfunction due to edentulous atrophic ridges. Guided bone regeneration was performed with titanium meshes combined with autogenous bone grafting and heterologous bovine bone mineral grafting, and exclusively a “poncho technique” soft tissue approach for all the cases. After a mean 9 months of graft healing (range 6–12 months), titanium meshes were removed, and implant surgery was subsequently performed. The results we obtained were positive in terms of volumetric increases in height, length and thickness of the atrophic ridges without biological complications detectable before implant surgery. Results: Out of nine, one site met titanium mesh exposure: however, in all 10 sites a three-dimensional volumetric bone implementation was obtained. The statistical results were estimated by uploading and superimposing cbct scans before and after CBR surgery for each patient, so it was possible evaluate the maximum linear vertical and horizontal bone gain through dedicated Cad Cam software (Exocad GmbH^®^). The average horizontal gain was 6.37 ± 2.17 mm (range 2.78–9.12 mm) and vertical gain was 5.95 ± 2.06 mm (range 2.68–9.02 mm). A total of 18 implants were placed into the grafted sites with a 100% survival rate (clearly they are relative percentages to be compared to the short time elapsed). Conclusions: The results we obtained in this study suggest that this CBR procedure (Yxoss^®^ by Reoss) is reliable and safe for bone regeneration to allow implant-prosthetic restoration in horizontal, vertical and combined bone defects. The soft tissue management is diriment: all the cases were managed with a “poncho” flap approach to decrease exposure complication.

## 1. Introduction

Nowadays, it can be assumed that dental implantology represents a reliable treatment both aesthetically and in functional restoration according to the different edentulism stage [1]. However, the loss of dental elements commonly turns over into a lack of bone that prevents the implant rehabilitation of the affected areas.

The main objective of all regenerative techniques in the dental field is to stimulate new bone tissue so the correct inter arches prosthetic relationship could be easier than in the initial clinical situation [2]. Guided bone regeneration (GBR) is a well consolidated surgical procedure that allows new bone formation in atrophies or various types of hard tissue defects.

Titanium-reinforced polytetrafluoroethylene (PTFE) membranes held in place by small screws are considered the best equipment for tissue augmentation during or before implant insertion surgery (even without total and universal consent on the part of all clinical users) [3].

To date, there is no unique scientifically shared consensus concerning the most suitable membrane choice (non-absorbable or resorbable) [4].

The implant success on regenerated bone in the literature is postulated to be between 68% and 100% [5,6].

The traditional titanium mesh (TM) represents a good alternative tool to restore bone defects.

However, the literature reports some criticism concerning TM, such as a learning curve to acquire the skills to cut, shape and adapt the TM to the irregular defects. This procedure usually is an intraoperative time-consuming phase, and the final result might be unideal: angles and edges, if not properly rounded, could represent, in the healing phase, a traumatic aspect for the surrounding soft tissue. This condition may lead to flap perforation and TM exposure. The clinical consequence may be infection and partial or total loss of the grafted bone [7].

The digital flow has been an ally in integrating this TM technique and supporting it to overcome limitations and disadvantages.

A new generation of titanium meshes has been recently introduced: these digital concepted titanium meshes are Cad Cam designed and created to be customized and precisely adapted to an irregular bone defect. Cad Cam technology allows one to produce laser sintered three dimensional custom scaffolds [8,9].

This clinical case series study focuses on the clinical efficacy of digital custom titanium mesh combined with autologous bone and bovine bone minerals (BBM) covered with collagen membranes to restore atrophic ridges. Specifically, it underlines the horizontal and vertical bone gain (digitally determined) in association with the peculiar poncho technique approach for the soft tissue management, which has been perfected in recent years, especially by the Veronese school. It allows one to avoid tension around the flap. The packaging of the flap according to this methodology is perhaps the most delicate and important point for a good result of the entire intervention, which is why it must be performed in the best way with the utmost care and precision with respect to the adjacent tissues.

## 2. Materials and Methods

This case series enrolled nine patients referred to the Maxillofacial Surgery and Dentistry Unit of the Hospital G.B. Rossi in Verona to solve chewing and functional problems due to atrophic edentulous ridges. All of them met the following inclusion criteria:

1. Clear clinical and radiographic identification (using some or all the tools at our disposal) of maxillary/mandibular bone defects or atrophic ridge (different in causality but similar for problems presented), specifically less than 8 mm in height and 5 in thickness.
2. No presence of any local or systemic contraindication to surgical treatment, such as infections, smoking habit of >10 cigarettes a day, uncontrolled diabetes (HBA1c ≥ 7.5%), previous radiotherapy in the head and neck anatomical areas, chemotherapy, liver, blood and kidney diseases, immunosuppression, state of pregnancy, inflammatory and autoimmune diseases of the oral cavity, poor oral hygiene and poor motivation.
3. No (learned from clinical practice) need to insert dental implants before 8 months of complete healing.
4. Nine patients (3 males and 6 females) aged between 43 and 65 years (mean 53.11 years, SD 6.79) affected by oral dysfunction due to atrophic edentulous ridges were enrolled in this study.

Table 1 reports the details concerning the distribution of the bone defects.

All of them received a customized titanium mesh (Yxoss CBR^®^ Reoss) derived from a Cad Cam technology and necessary to maintain in the grafted area a mixture of autogenous bone particles and deproteinized bovine bone mineral (Bio-Oss^®^—Geistlich Biomaterials AG, Wolhusen, Switzerland) in a 50% ratio, and it was covered with collagen membranes (Bio-Gide^®^—Geistlich Biomaterials AG).

Informed consent was obtained from all patients prior to treatment, including (a) the purpose of the treatment; (b) a detailed description of the procedure; (c) the potential side effects and complications; (d) the patients’ authorization to use their data for statistical analysis.

The study was conducted in accordance with the Declaration of Helsinki, and the protocol was approved by the Ethics Committee of the University of Verona, Italy (Project identification code CICG-1935CESC, date: 16 October 2018).

Preoperative planning and patient preparation.

**Figure 1:**
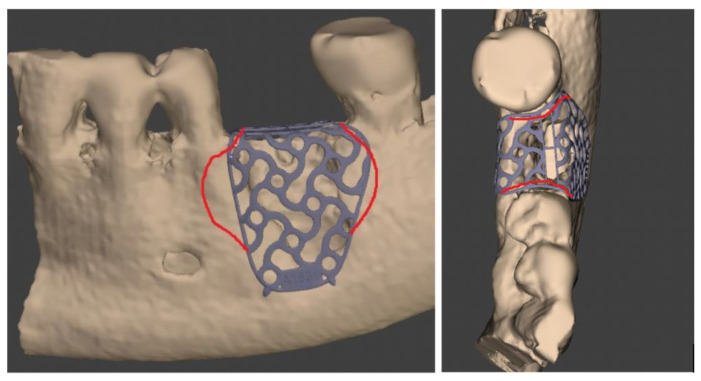
These screenshots show all the customized Ti mesh changes shared between the surgeon and the digital technician in the pre-clinical digital planning.

All the enrolled patients received a CBCT scan before CBR (T0): DICOM files obtained were sent to the Master Centre (Yxoss CBR^®^ by Reoss) to share the Ti mesh planning and design. A digital model of the Ti-mesh was provided by the manufacturer for the final approval. Any planned change to the mesh, if necessary, was communicated by clinician to the digital technician to achieve an appropriate mesh morphology (Figure 1).

These CBCTs were compared as the reference point (time 0 = T0) to the following CBCTs (T1) to calculate bone volume gain.

After final clinician approval, the custom Ti mesh (class IV titanium) was produced through laser sintering CAD/CAM technology (Yxoss CBR^®^ by Reoss).

The treatment protocol was as follows:

Antibiotic prophylaxis, oral administration of amoxicillin and clavulanate (2 pills, 1 g, 1 h before the surgical date), was prescribed.

**Figure 2:**
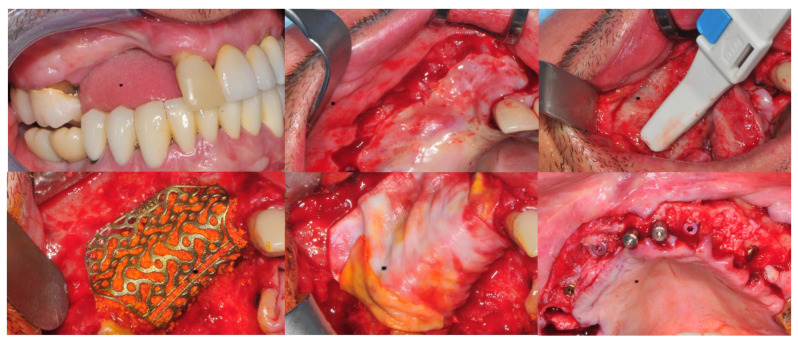
CBR involving upper jaws. Top line, from left to right: initial clinical situation, flap design, bone refreshing. Bottom line from left to right: CBR in place, covering of it with a resorbable membrane, implanting of insertion after grid removal and clinical evaluation of the bone quality obtained.

**Figure 3:**
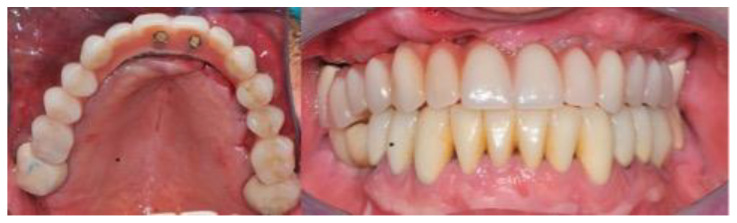
Occlusal and frontal intra-oral aspects of the immediate loaded full arch prosthesis. (**Left**): occlusal view. (**Right**): front view.

- Anesthesia with loco-regional infiltration of anesthetic mepivacaine with adrenaline 1:100,000 (no patient had physio-pathological conditions to require anesthesia without constricting vessel liquid).
- Flap design: Very gentle soft tissue handling had to be carried out. The shape and extension of the flap had to ensure no tension in primary closure, especially with a bulky graft. Our suggestion in these cases is the “poncho” flap [8,10]. This technique includes a first large vestibular incision of the mucosa and the muscle/periosteal structures to increase their mobility, and two further vertical incisions not so close to the graft site. After the incision, the preparation of a muco-periosteal flap and the remotion of scar tissue, a full thickness flap was raised until the bone defect was uncovered [11,12,13]. Finally, the positioning of the customized titanium mesh was passively tested to evaluate its fit intra-operatively.
- Preparation of the receiving site: It was necessary to carefully clean the underlying bone and drill corticotomy to allow bleeding and to make a connection with the inner medulla bone, which has osteogenetic potential.
- Particulate bone: We used miscellaneous bone, autologous and heterologous, mixed together in equal parts (50:50) (Bio-Oss^®^—Geistlich Biomaterials AG). The autologous bone samples (essential to allow the 50:50 mix) were collected from different sites based on volumetric availability; they included the retromolar mandible area, tuber maxillae and ascending branches of the mandible (by combining all the quantities from the various sites for a total of approximately 3.5–4 cm^3^). We used a manual bonescraper (Manufactured from AISI 420 German stainless steel. Product Conforms to ISO 9001, CE-Quality Mark, ISO 13485).
- Positioning of the customized titanium mesh (Yxoss CBR^®^ Reoss): since the titanium grid was built on previously designed projects, it was not necessary to make any changes because it fit perfectly to the bone residue.
- Fixation and coverage: Fixation was a fundamental point for the success of the intervention. Usually, mini titanium screws are used (5 mm long and 1.35 mm diameter), placed with special dedicated screwdrivers and subsequently covered with a resorbable membrane (Bio-Gide^®^—Geistlich Biomaterials AG).
- Passivation of the flap: Subtle periosteal incisions were performed to allow easier and greater mobility of the entire flap aimed at closure by primary intention without any kind of tension, thereby creating passivation.
- Suture: Precise closure had to be obtained through horizontal mattress sutures 5 mm beneath from the incision line, and subsequently, with single interrupted stitches connecting the edges of the flap. In this way it was possible to obtain an approximation of the edges of the flap with intimate contact between the two parts and the interposition of connective tissue (Figure 2 and Figure 3)

Oral antibiotics were prescribed for all the patients (amoxicillin and clavulanate-3 g/day for 6 days). Sutures were removed 2–3 weeks after surgery.

During the healing phase, only one patient had later exposure of the titanium mesh: it did not affect the whole CBR procedure because partial removal of the exposed titanium mesh was performed: the poncho technique was re-applied, including sectioning with rotary cutter of the portion of the exposed grid and repositioning of the cover flap.

**Figure 4:**
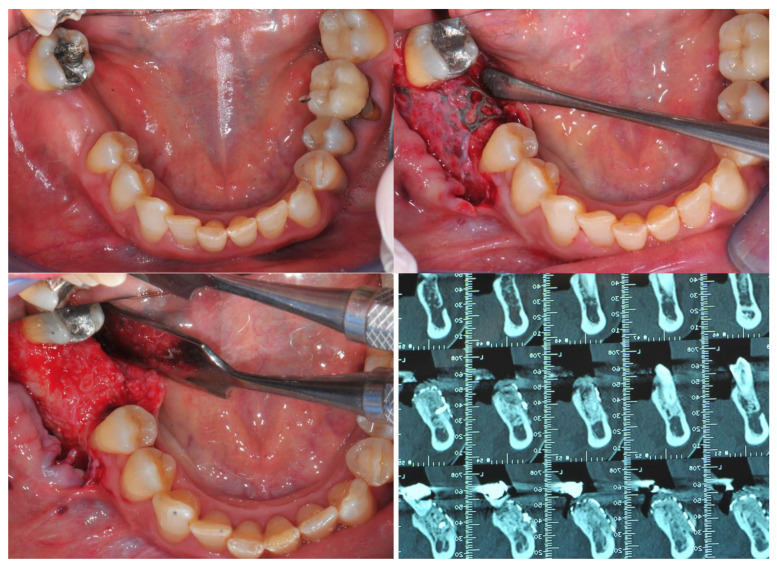
This composite resumes the surgical workflow from the custom Ti Mesh graft healing to the implant placement. In the CBCT (T1) screenshots it is possible to appreciate the bone volume gain. From the top left clockwise: starting situation, grid removal after 6 months; section of CBCT shows the bone volume gain, occlusal view just before implant surgery.

**Figure 5:**
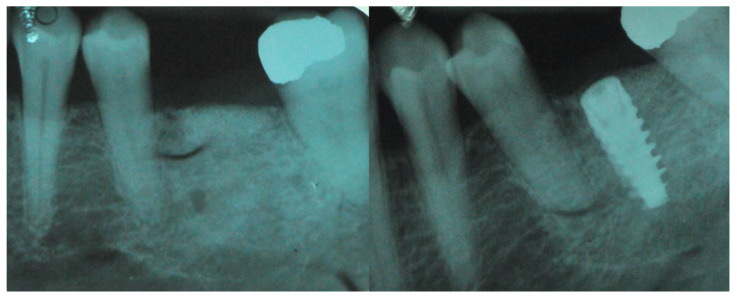
Intra oral radiograph before and after implant placement. Left: intra-oral X-ray after grid removal and healing occurred. Right: fixture installation.

Not less than six months after CBR, a CBCT was scanned for all the patients (T1) to control the outcomes of the surgery after the graft healing phase (6–12 months) (Figure 4 and Figure 5).

It was possible to compare the Dicom file derived from T0 e T1 CBCT scan exams to evaluate all these CBR procedures and the bone gain from a bi/tridimensional aspect.

First of all, through dedicated software (Real Guide Universal Open System 3 Diemme), all the Dicom format files were converted to .stl format files (known as a segmentation process).

**Figure 6:**
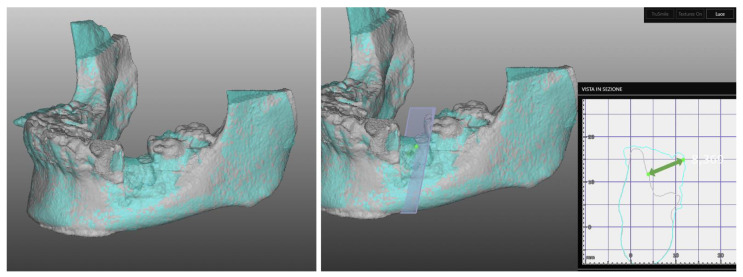
Segmentation of Dicom file and (T0–T1) CBCT superimposition to evaluate horizontal, vertical and bone volume gains.

**Figure 7:**
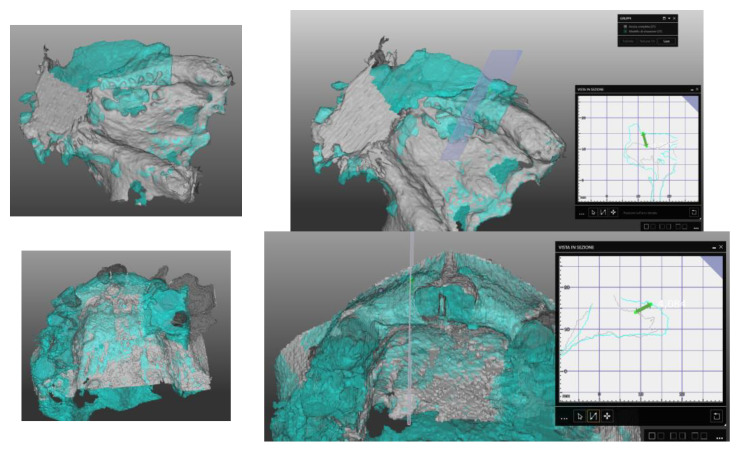
Segmentation of Dicom file and (T0–T1) CBCT superimposition to evaluate horizontal, vertical and bone volume gains for upper jaw case.

After that, through other dedicated software (Exocad GmbH-specific tool “cross section view”), it was possible to superimpose T0 e T1 CBCT scans to trace and evaluate the linear maximum vertical and horizontal gains for all the patients (in mm) and bone volume gains for all the grafted sites (in mm^3^) (Figure 6 and Figure 7).

All the values collected are reported in Table 1.

## 3. Results and Statistical Analysis

### Statistical Analyses

**Figure 8:**
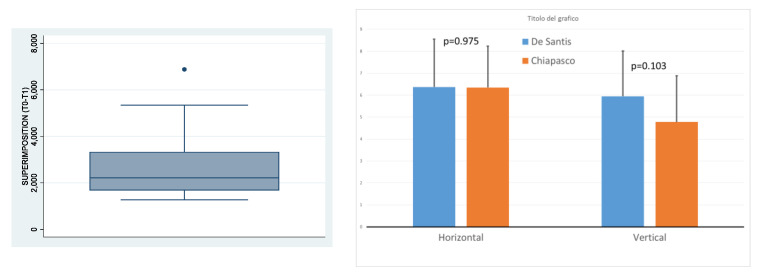
Maximum and vertical gains, obtained in the present series, are compared with historical data, and the significance of differences was evaluated by the *t*-test for unpaired data, after verifying the homoscedasticity and normality assumptions.

Qualitative variables are summarized by absolute and percent frequencies, and quantitative variables by mean and standard deviation (Figure 8).

Maximum and vertical gains, obtained in the present series, were compared with historical data, and the significance of differences was evaluated by the *t*-test for unpaired data, after verifying the homoscedasticity and normality assumptions (Figure 8).

Overall, 18 implants were inserted in nine patients. Four of them were put in mandibular sites and five in maxillary sites. The resulting total healing took place in 8–9 months for eight patients, but not in one case due to limited mesh exposure (in a maxillary site). In no case was the bone volume gain effected.

In this last case, only the exposed portion of the custom titanium mesh was removed, and this patient used chlorhexidine-based (0.2%) mouthwash.

Despite the limited number of the sample, all 18 implants (placed at T1 after the custom titanium mesh removal) met osteointegration and showed a survival rate of 100%.

## 4. Discussion

First of all, in this context we should have a little digression about the use of bio-material fillers in this surgical CBR technique.

In the last decades, the literature concerning the deproteinized bovine bone has been extensive and increasing [11,12,13,14,15]. It suggests several and undoubted advantages, such as the potentially infinite quantity of material that can be used and the absence of further and more complex surgical interventions to obtain it. Another aspect to be considered, is the fundamental and intrinsic property of osteoconduction [16,17,18] (defined as the process by which the regeneration of new bone tissue is facilitated, acting as a temporary scaffold); the autologous bone, which is universally recognized as the gold standard for bone reconstructions, adds osteoinduction [19,20,21,22] (the ability to stimulate the cells proposed to form bone by inducing or increasing their function precisely with the so-called growth factors and morphogenetic proteins induce the formation of bone) and osteogenesis [23,24,25,26] (the osteoblasts mediated process), which leads to the formation of new bone tissue starting from the graft.

These three characteristics listed above are essential cornerstones for guided bone regeneration. Thus, this 50:50 mix can well be considered the biomaterial par excellence. It is defined by “Definitions in Biomaterials”, Chester consensus conference in 1986 [27], as any substance intended to be connected to living tissues and/or biological fluids to maintain or change the shapes or to replace any tissue, organ or function of the body and further demonstrating, according to Osborne, three essential characteristics of a biomaterial, or rather biotolerance, bioinertness and bioactivity [28].

Using a resorbable membrane of collagenic nature to improve healing is universally recognized to be faster and better [29,30,31,32,33]. The fundamental principle of GBR (guided bone regeneration) is in fact the use of this type of membrane to maintain primary wound closure, angiogenesis, space creation/maintenance and stability for the initial blood clot [34]. In addition, it seems that the interaction between the titanium mesh and the resorbable membrane can help the formation of a soft tissue similar to the periosteum [35] that can be formed on the internal or external surface of the mesh, or both sides of the mesh, and promote mucosal healing [36], protect titanium mesh and bone graft material from gingiva and reduce inflammation during wound healing [37]. Ultimately, we recall that a negative predictive factor in the success of the use of this technique is the lack of soft tissues enveloping the area. Proximity to muscle insertions and a lack of keratinized mucosa are factors that affect the mobilization of the flap, and therefore, increase the risk of dehiscence [7]. Therefore, the use of this tissue identified histologically by Arx could be useful for compensating the physiological lack of keratinized tissue.

The last but not the least biomaterial to be mentioned is titanium, which is widely used in surgical treatments due to its high stiffness, low density, corrosion resistance and effective biocompatibility. Titanium mechanical properties are fundamental to a safe CBR strategy: high strength and stiffness concur to space maintenance in the osteogenesis; the stability is mandatory for the bone graft volume during wound healing [38]. Thickness and porosity are mechanical features affecting titanium properties. Literature suggests that the thickness of titanium is a factor affecting the final bone formation; moreover, the pore diameter may affect the proportions of soft and hard tissue under the titanium mesh [39]. The thickness of titanium mesh (commonly ranging from 0.1 to 0.6 mm) is proportionally linked to its mechanical properties.

A titanium mesh of 0.2 mm is suitable for most instances. At this thickness, titanium mesh can provide sufficient stiffness to maintain space and protect grafts, while offering appropriate flexibility to reduce the risk of tissue rupture. Concerning the pore diameter, it also affects the performance of titanium mesh during bone augmentation. The pore of the titanium mesh seems to rise an essential role in establishing blood supply and facilitating metabolic processes of the grafts at the defect site [40].

This aspect may be associated with the increased delivery of blood supply, nutrients and oxygen due to the large aperture. On the contrary, a study showed that the use of a titanium mesh with large diameter (>2 mm) may lead to more soft tissue growth upon the surface of new bone than the use of a titanium mesh with a small diameter [41]. However, the relationship between the pore size of titanium mesh and bone formation is still controversial. For the intrinsic nature of pores on titanium mesh, selective cell isolation is not insured, and soft tissue often grows under titanium meshes [40].

### 4.1. Biological Properties and Osteogenic Property

Titanium mesh offers good biocompatibility. This behavior of materials usually can be distinguished into corrosion resistance and cytotoxicity. Commonly, titanium shows low electrical conductivity; it is basically disposed to perform electrochemical oxidation to form a passive and inert oxide layer [42]. This oxide layer can be retained at the pH of the human body, leading to high and persistent corrosion resistance for titanium [43]. A thin layer of 1–2 mm thick soft tissue (defined as “pseudo-periosteum”) has been observed in several studies upon the regenerated bone surface through a titanium mesh [35].

Probably, the pseudo-periosteum formation could depend on the not sufficient cell exclusion behavior of the titanium mesh due to its pores. A clear set of evidence sustains its role in bone graft protection, and its roles in infection prevention and absorption. Currently, it is often removed with the titanium mesh in a subsequent operation [44].

### 4.2. Progress in Material Modification

In the last few decades, the research trend has been developing surface modification of titanium mesh to achieve better biological activity. Bioactive coatings for accelerating bone regeneration by improving the differentiation and proliferation of osteoblasts have been widely developed in tissue engineering.

In a previous study conducted on a rat model, Nguyen compared the different behavior of titanium mesh covered by calcium phosphorus coating and untreated titanium mesh [45,46,47].

There was no soft tissue intervention under the titanium mesh of the experimental group, and the bone density was higher (*p* < 0.05). There was consequently improved structural durability of the grafted site. In another succeeding study, strontium was proposed to promote osteoblasts’ proliferation and differentiation, and inhibit osteoclasts’ activity.

It was tested in guided bone regeneration in a rat model combined with a calcium–phosphorus coating on the surface of titanium mesh. Compared to no treatment and calcium–phosphorus-coated titanium mesh treatment, the strontium–calcium–phosphorus coating resulted in more uniform and denser bone, enabling higher bone density in the regenerated area, improving the osseointegration between bone and titanium mesh and preventing infiltration of soft tissue in the early stage of healing.

After this study it is reasonable to think that proper coating treatment can lead to more stable bone regeneration without specific adverse reactions.

### 4.3. Radiological Considerations

Another aspect we should underline is the radiological behavior of customized titanium mesh during the cbct or rx scan: its physico-chemical features allow it to minimize the scattering effect. This implicitly guarantees a clear radiological image of the control exam (T1), and it is thus possible to trace the calculation lines underlying the edge of the customized mesh, thereby minimizing the error. Customized titanium meshes have been recently proposed as an alternative surgical procedure to the conventional TM. [8] A lot of advantages seem to sustain this affirmation:

(a) The custom titanium meshes features do not need modelling or trimming;
(b) These custom scaffolds are self-contained, and it is possible to load the graft outside the oral cavity, causing very strong reductions in the risks of graft dispersion and contamination intraorally;
(c) Safe stabilization with a small number of screws and custom mesh passiveness.

All these aspects seem to be so relevant for safe graft healing below the custom titanium mesh.

To the best of our knowledge [7,8,9], the main critical issue concerning TM is mesh exposure (at different time) with variable degrees of bone resorption.

This aspect seems to depend on a strong learning curve to acquire the skills to cut, shape and adapt the TM to the irregular defects. This procedure usually is an intraoperatively time-consuming phase, and the final result might not be ideal: angles and edges, if not properly rounded, could represent, in the healing phase, a traumatic aspect for the surrounding soft tissue. This condition may lead to flap perforation and TM exposure. The clinical consequences may be infection and partial or total loss of the grafted bone [7].

In the only case of exposure of the grid, it was not necessary to remove it, as there were no signs of infection, and it did not worsen or diminish the pre-established final result of bone regeneration. This exposure occurred in the maxilla, a result that might be in accordance with other authors using the same location in the mouth [8], but in this case we think it may have been due to the friction of the grid (particularly extended) with the overlying soft tissues.

All the authors believe that soft tissue management represents the most critical issue for this technique, which involves the shape of the surgical flap, for a stress-free first intention closure. The extension of the incision should be broad, involving at least two additional dental elements both medially and distally to allow a more secure and sealed closure.

For all the nine patients, a “poncho technique” soft tissue approach was performed: this soft tissue management seems to a play a favorable role in preventing meshes’ exposure, as confirmed by other authors, compared to the crestal incision approach [8].

The presence of a fibrous granulation tissue below the titanium mesh could increase the low probability of infection. This tissue has been analyzed and is similar to the periosteum, constituting a real mechanical shield from bacterial contamination and subsequent surface exposure with bone reduction. The real nature and origin of this tissue are not well known [48,49,50].

The present clinical case series has shown the potential of the digital flow to integrate a conventional surgical procedure such as TM to achieve a safer and more efficacious bone regeneration technique.

Results from the present study seem to confirm data reported in literature: an average horizontal gain of 6.37 ± 2.17 mm (range 2.78–9.12 mm) and a vertical gain of 5.95 ± 2.06 mm (range 2.68–9.02 mm). In detail, if these mean values are compared to those obtained by other authors, we notice: 0.9747 in horizontal gains, and t: 0.1030 in vertical gains [33,51].

Furthermore, other results derived from the data collection concerning the T0–T1 CBCT scans’ superimposition have confirmed a mean bone volume gain value of 3012 ± 1938 mm^3^ (range 1273–6879). It is, however, worth nothing that these last results gave us an overestimated mean bone volume gain value if compared to the presurgical planned bone volume gain value. Results are also comforting if related to other GBR procedures with resorbable and non-resorbable membranes [33].

## 5. Conclusions

Given the small number of implants inserted and the even smaller number of patients enrolled for these treatments, we are now far from standardization of procedures and a global consensus. However, this study suggests that CBRs with customized meshes represent predictable and encouraging alternatives for the future.

The planned construction of the grid itself and the attention paid to the design of the flap and its management are essential factors for clinical success. Partial exposure of the mesh does not necessarily lead to the failure of the CBR technique. All authors consider the surgical “poncho” technique the best approach to avoid uncovering of the grid, superinfections and the failure of the intervention itself in general. In addition, careful pre-operative planning and the precision of the grid/bone tissue fit seem to further reduce the margins of operator-dependent error. 

## Figures and Tables

**Table 1:** Distribution of the bone defects and maximum vertical/horizontal/volume bone gain recovered through measurements on CTCB.

Patient No.	Site	Age	Gender	Max Horizontal Gain (mm)	Max Vertical Gain (mm)	Pre Clinical Volume Gain Plan (mm^3^)	Superimposition (T0–T1)
1	36	65	F	4.4	4.7	718	3330.56
2	36	51	F	9.1	9.0	927	3221.47
3	22–23–24	43	F	6.0	5.5	1008	1273.18
4	13–11–23–24	62	F	5.7	4.5	1208	2960.2
5	14–15–16	49	M	8.6	7.3	4250	5342.31
6	46	55	M	5.2	4.9	364	1686.84
7	13	53	M	6.7	6.3	1070	6879.74
8	36	51	F	8.9	8.6	895	2217.1
9	11–12–13–14	49	F	2.8	2.7	1632	1676.75

## Data Availability

Not applicable.
